# Artificial intelligence in healthcare: rethinking doctor-patient relationship in megacities

**DOI:** 10.3389/frhs.2025.1694139

**Published:** 2025-09-24

**Authors:** Qi Chen

**Affiliations:** College of Applied Arts and Science, Beijing Union University, Beijing, China

**Keywords:** artificial intelligence, healthcare, doctor-patient relationship, megacity, Beijing

## Abstract

**Introduction:**

Artificial intelligence has been extensively applied in healthcare, offering significant potential to improve the quality of medical services. However, it also introduces critical challenges, such as privacy infringement, algorithmic discrimination, and ambiguous liability. The integration of artificial intelligence inevitably influences the doctor-patient relationship, which is pronounced in megacities. This study aims to explore the application of artificial intelligence in megacity healthcare system, exam the multidimensional transformation of the doctor-patient relationship and propose new governance frameworks.

**Methods:**

This study examines how artificial intelligence can effectively address systemic challenges within megacity healthcare systems while leveraging technological and institutional advantages to maximize its benefits, with a focus on Beijing as a primary case.

**Results:**

The integration of artificial intelligence inevitably influences the doctor-patient relationship, reducing information asymmetry, enhancing patient autonomy, and transforming the traditional doctor-patient dualistic interaction structure into a doctor-artificial intelligence-patient triad interaction structure. These effects are pronounced in megacities, presenting new challenges including crisis of trust, intensified disputes, and emotional and communication distance.

**Discussion:**

Given that the integration of artificial intelligence into healthcare is inevitable, especially for megacities like Beijing, proactive governance is essential. This includes institutionalizing the triad interaction model, deepening the integration of artificial intelligence in healthcare by leveraging the advantages of megacities, and establishing regulatory frameworks to mitigate risks while harnessing potential.

## 1 Introduction

Since its inception, artificial intelligence has rapidly transformed the world. Encompassing a wide variety of fields, artificial intelligence is often ranked as one of the most interesting and fastest-growing disciplines (1). It has already been integrated into various areas of healthcare, and is rapidly re-engineering this domain, with one “legendary Silicon Valley investor” even arguing that robots armed with artificial intelligence will replace doctors by 2035 (2).

The doctor–patient relationship has been the primary foundation of medicine since its beginnings (3). This professional relationship is of great significance due to its influence on diagnostic accuracy and treatment effectiveness (3), as well as on the health and stability of society as a whole. The doctor–patient relationship has undergone continuous transition with medical and sociological developments (4). The integration of artificial intelligence in healthcare inevitably promises a profound impact on this relationship, necessitating critical scholarly attention and in-depth analytical reflection.

According to the United Nations report on urbanization, alongside the growth of the global population, population ageing, and international migration, urbanization constitutes one of four demographic mega-trends (5). Megacities, with populations exceeding 10 million, are a typical manifestation of urbanization. They are of extreme importance due to the scale of their populations and their tendency to be centers of economic, political, and technological power (6). The application of artificial intelligence in healthcare offers critical solutions to systemic challenges confronting megacity healthcare systems. However, the obstacles to integrating artificial intelligence in healthcare are amplified due to the inherent complexities of megacity, which impact the doctor–patient relationship. This study systematically explores the application of artificial intelligence in megacity healthcare system by conducting a case analysis of Beijing, examining the multidimensional transformation of the doctor–patient relationship and proposing new governance frameworks.

## 2 Artificial intelligence in healthcare

Artificial intelligence is defined as a “general term for imitating human intelligence with computer systems” (7). It has been integrated into different domains, with its application in medicine including both virtual and physical branches (8). The virtual branch manifests as machine learning and deep learning, which use mathematical algorithms to help computers learn from data and experience (8). The physical branch encompasses medical equipment and the sophisticated robots being used in medical and health services (8). The application of artificial intelligence is revolutionizing the healthcare industry, presenting transformative potential alongside emergent challenges.

### 2.1 Application of artificial intelligence in healthcare

With continuous advancements, artificial intelligence has become embedded in every area of healthcare. For example, artificial intelligence has been applied in the fields of diagnosis and clinical care. Research shows that artificial intelligence has been successfully used to facilitate the detection of breast cancer, brain cancer and hybrid disease (9). By integrating machine learning and deep learning in medical imaging, precise and efficient disease detection and diagnosis can be achieved (10). In the field of surgery, robotic surgery is being utilized across multiple specialties, spanning general, gynecological, urological, cardiac, orthopedic, and head and neck surgery (11).

Additionally, artificial intelligence can provide full life-cycle health management. The application of artificial intelligence extends beyond diagnosis and clinical care to disease prevention, nursing care, and rehabilitation enhancement. Studies have suggested that artificial intelligence, especially machine learning, can predict the occurrence of individual chronic diseases (12) and facilitate health promotion and behavioral changes (13). By virtue of the capacity of artificial intelligence to process and interpret extensive biomechanical and physiological data, wearable technologies equipped with sensor and artificial intelligence capabilities enable healthcare professionals to design personalized rehabilitation programs (14).

In addition, artificial intelligence transforms the management of healthcare institutions. Its implementation is pivotal for handling extensive healthcare information, enhancing administrative workflows, optimizing resource allocation, and improving many other facets of hospital management (15). Artificial intelligence plays a similarly critical role in fields such as pharmaceutical research and outbreak response, among others (16, 17).

### 2.2 Advantages and challenges of artificial intelligence in healthcare

Artificial intelligence has the potential to enhance healthcare outcomes significantly. First, the integration of artificial intelligence can improve diagnostic precision. Studies have demonstrated that neuroimaging techniques enhanced by artificial intelligence could enable the precise detection of Alzheimer’s disease biomarkers in the early stage of diagnosis (18). Furthermore, the application of artificial intelligence could help to alleviate physician burnout, thereby reducing medical errors and improving the quality of healthcare, which can otherwise be poor in the context of burnout. By using the Complementarity-Driven Deferral to Clinical Workflow (CoDoC) system in breast cancer screening, artificial intelligence reduced clinician workload by 66% (19). Artificial intelligence could also optimize the allocation of medical resources, and telemedicine could transcend geographic barriers, enabling more people to access quality healthcare services. One study revealed that telemedicine achieved a cure rate of 84.8% for hepatitis C, significantly outperforming the 34.0% rate in referral (20).

While artificial intelligence has the potential to significantly enhance healthcare outcomes, it also presents substantial challenges. Advanced artificial intelligence models rely heavily on extensive, high-quality data, which raises concerns about data collection and patient privacy (21). Not only can an extensive amount of data describe an individual's health, but it can also identify the person. Given the potential risk of data breaches, patient information protection assumes particular significance. Algorithmic discrimination also presents a challenge when implementing artificial intelligence in healthcare. Three primary factors contribute to algorithmic discrimination: measurement errors arising from incomplete or incorrect data, selection bias due to under-inclusive or skewed training samples, and feedback loop bias, which perpetuates historical discrimination patterns (22). Algorithmic discrimination may lead to certain groups being subjected to differential treatment (23) and may even result in life-threatening consequences (24). Determining liability for artificial intelligence applications in healthcare presents another significant obstacle. Due to algorithmic opacity and the absence of recognized legal personhood, it is difficult to assign responsibility for medical injuries caused by artificial intelligence applications (25).

## 3 Artificial intelligence application in megacity healthcare systems

Global cities are becoming larger, with megacities experiencing the fastest growth rates (26). The urban scale effects of megacities amplify both the challenges confronting healthcare systems and the transformative potential of artificial intelligence. As a quintessential megacity and China's capital, Beijing's healthcare system faces significant issues, yet its unique position provides opportunities of optimization through artificial intelligence.

### 3.1 The challenges of Beijing's healthcare system

As a megacity with more than 20 million permanent residents (27), Beijing's healthcare system is under immense pressure. As well as its large permanent population base, the rate of population aging in Beijing is also higher than the national average. In 2023, the city housed 4.948 million citizens aged 60 and above, accounting for 22.64% of its permanent residents (28). Additionally, Beijing's population has high expectations for medical care. The city's employed inhabitants are well-educated; in 2022, 65.3% of residents had a higher education, 41.2 percentage points above China's national average and 11.6 percentage points ahead of Shanghai, which is in second place (29). Beijing residents also demonstrate superior health literacy, with the city's health literacy rate reaching 44.6% in 2024, surpassing the national average by 12.73 percentage points (30, 31). Compounding this, Beijing's healthcare system faces a substantial volume of non-local patients. In 2022, patients from other provinces seeking care at Beijing's tertiary hospitals accounted for 12.80% of all interprovincial patients in China, ranking second nationally (32). This enormous influx of non-local patients, combined with Beijing's massive population base, higher-than-national-average aging rate, and the high health literacy of residents, drives large-scale, complex, and quality-sensitive healthcare demands, placing immense pressure on the system.

Additionally, Beijing concentrates a disproportionate share of premium medical resources, which has resulted in chronic overburdening at certain hospitals. This manifests as appointment shortages, excessive clinician workloads, and sustained high-pressure on the system. More than 80% of Beijing's top-tier hospitals, with their superior resources and talent, are located in the central urban area (33). Conversely, medical and health institutions at the community level have relatively weak infrastructure, uneven talent pools, and varying technical capabilities (33). This disparity has created a prominent “siphoning” effect, with patients attending large hospitals in the central urban area, while community institutions are not very attractive to residents. This effect also applies to bed utilization efficiency. Due to patient distrust stemming from gaps in technological resources and deficiencies in highly skilled health professionals, the bed utilization efficiency of community health service centers is lower than that of general and specialized hospitals (34).

As China's political, cultural, international, and technological hub, Beijing faces unique challenges in its public health emergency management system. It experiences high population mobility and frequent international and domestic interactions. According to the Center for International Exchanges Index 2024 report, Beijing ranks seventh globally for international exchanges, being the only city on the Chinese mainland that makes the top ten (35). In 2024 alone, the city received 3.942 million international and 370 million domestic tourists (36). This creates complexities and difficulties for rapidly identifying public health emergencies, making the surveillance and early warning of infectious diseases challenging and straining emergency resource allocation.

### 3.2 The feasibility and advantages of artificial intelligence in Beijing’s healthcare system

The application of artificial intelligence in healthcare could effectively address the challenges that Beijing's healthcare system is facing. Internet hospitals, telemedicine, and online consultations can effectively enable patient diversion, significantly reduce patient flow through hospitals with highly concentrated high-quality medical resources, and provide patients with convenient access to improved medical services. Furthermore, other artificial intelligence developments in the fields of clinical decision support and medical imaging interpretation can reduce cognitive load and procedural burdens for healthcare providers, thereby mitigating occupational stress. In the public health area, artificial intelligence empowers capabilities across risk prediction and communication, public health surveillance, disease forecasting, and infodemic management (37, 38).

Beijing has prioritized developing artificial intelligence in healthcare, implementing many applications across healthcare services. By 2020, eight hospitals in Beijing had already launched online diagnosis, treatment and drug delivery services (39). This was recognized by the National Health Commission, which issued a circular calling on hospitals to advance the development of online diagnosis and treatment, as well as internet-based hospitals, to ease the pressure on outpatient clinics (39). In 2021, the Internet Hospital of Peking Union Medical College Hospital (PUMCH) was approved by the Beijing Municipal Health Commission as the first Internet hospital in the city (40). This hospital provides follow-up services online, including online consultation, online examinations, the ability to access in-hospital medical records, and others (40).

As China's center for technological innovation, Beijing possesses distinctive technical advantages. The city is home to 92 colleges and universities, more than 1,000 research institutes (41), and the highest number of high-tech and unicorn enterprises in the country (41). In 2023, Beijing's R&D intensity reached 6.73%, surpassing China's national average and ranking first nationally (42). According to the Nature Index, Beijing has consistently ranked first in the global science city rankings for eight consecutive years (41). Leveraging these technological strengths, the city has demonstrated competitive advantages in the integration of artificial intelligence within its healthcare system. In 2025, Tsinghua University introduced the AI Agent Hospital, which “aims to create a closed-loop ecosystem of ‘AI + Healthcare + Education + Research’, enhancing the efficient expansion and equitable distribution of high-quality medical resources” (43).

Furthermore, Beijing promotes the integration of artificial intelligence into healthcare services through policy frameworks. In 2025, the city released Beijing Action Plan for Accelerating the Innovative Development of Artificial Intelligence + Medical Health (2025–2027), which aims to transform Beijing into an internationally influential hub for innovation in artificial intelligence and medical health (44).

The case of Beijing highlights the imperative for megacities to integrate artificial intelligence into their healthcare systems and demonstrates the advantages of these developments. However, it also amplifies the challenges of applying artificial intelligence in healthcare, especially in terms of the way it transforms the doctor–patient relationship.

## 4 Transformation of the doctor–patient relationship

The doctor–patient relationship has evolved throughout history. Both doctors’ and patients’ capabilities in terms of self-reflection, communication, and technical skills, as well as the socio-political and intellectual-scientific climate, all influence this crucial relationship (4). In 1956, Szasz and Hollender introduced the three basic models of doctor–patient relationships, which are: (i) active–passivity, (ii) guidance–cooperation, and (iii) mutual participation (45). In the active–passivity and guidance–cooperation models, doctors are in a dominant position, whereas the mutual participation model places a greater emphasis on patients. With the emergence of the bio-psycho-social medical model and increasing awareness of patient rights, the mutual participation model is regarded as an ideal model for the doctor–patient relationship. The doctor–patient relationship was traditionally framed in terms of benevolent paternalism; with the emergence of the Internet, patients have begun to take more initiative with their healthcare (46). The application of artificial intelligence in healthcare has further transformed the doctor–patient relationship.

### 4.1 Bridging information asymmetry between doctor and patient

Information asymmetry constitutes a fundamental characteristic of the doctor–patient relationship. Being a member of the medical profession requires extensive knowledge acquisition and prolonged training. However, given the inherent breadth and complexity of medical science, even professionally trained doctors struggle to attain comprehensive expertise in all areas of medical practice. For patients, it is almost impossible to acquire the same medical knowledge as doctors. Additionally, a patient's medical history, medication habits, lifestyle, dietary patterns, physical condition, psychological state, living environment, and other factors are closely associated with disease prevention and diagnosis. However, much of this information is of a private nature, and without the patient's full cooperation, it is difficult for doctors to gain a comprehensive understanding of it.

The integration of artificial intelligence can bridge information asymmetry between doctor and patient. With the support of artificial intelligence technology, accessing medical information has become highly convenient. It is now common for people to seek medical information using apps or generative artificial intelligence. Studies have found that the trend of online information-seeking behavior empowers individuals to enhance their knowledge about health concerns and symptoms (47). Likewise, it is now much easier for doctors to monitor patients remotely and access patient data. For example, HeartGuide is a blood pressure monitor that users wear to track their blood pressure and its results can be shared with doctors (48).

### 4.2 Advancing patient autonomy in decision making

The realization of the patient autonomy principle depends on the following three rights: (i) the right to privacy or the right to intimacy, (ii) the right to health information and (iii) the right to decide on matters concerning one's own health (49). Respecting patients’ autonomy is one of the fundamental principles of medical practice (50). Historically, medical knowledge was exclusively held by doctors, and patients had limited access to health information, meaning that the doctor–patient relationship remained doctor-dominated. Patients’ medical decision-making rights were constrained by doctors, and they were expected to simply comply with doctors’ medical advice.

As discussed in Section 4.1, the integration of artificial intelligence into healthcare has made it much easier for people to look up medical information. This has prompted increasing numbers of individuals to conduct online symptom research before visiting the doctor. When these people finally go to see the doctor, they often arrive with preliminary self-diagnoses and may even question the doctor's medical advice. As the opinions and recommendations of artificial intelligence systems are given more weight, doctors’ influence and decision-making power are being challenged, strengthening patient autonomy.

### 4.3 Transcending the doctor–patient dualistic interaction structure

The doctor–patient relationship is a long-standing interaction between medical providers and their patients. However, with the integration of artificial intelligence in healthcare, this dualistic interaction structure is being broken. Artificial intelligence will be used in almost every type of healthcare in the future (51), inevitably introducing artificial intelligence as a third party in the doctor–patient relationship. Doctors use artificial intelligence to enhance diagnostic accuracy, reduce medical errors, and alleviate workflow burdens. Patients use artificial intelligence to access medical information and promote their own health. The traditional doctor–patient dualistic interaction structure has been transformed into a doctor–artificial intelligence–patient triad interaction structure.

### 4.4 Tensions of doctor–patient relationship in megacity contexts

Building upon the paradigmatic case of Beijing presented in Section 3, the confluence of demographic complexity, concentrated technological capital, and policy support in megacities has systematically amplified the transformative impact of artificial intelligence on the doctor–patient relationship.

The application of artificial intelligence in healthcare, while transforming traditional information asymmetry, has also somewhat exacerbated the doctor–patient trust crisis. In the traditional healthcare-seeking process, patients typically selected doctors based on institutional reputation and professional credentials, and then consult other doctors for further verification (52). As demonstrated in Section 4.1, it is now common for people to look up medical information using apps or generative artificial intelligence. Before the consultation, patients have already gained some understanding of their own conditions through the internet, generative artificial intelligence, and other channels (52). During the consultation, they are likely to compare their own understanding with the doctor's judgment to determine whether the doctor is credible (52). If the doctor fails to respond well to the patient, the patient's doubts about them may be raised. From the doctor's perspective, if they know that the patient has conducted pre-consultation research, they may develop heightened expectations regarding the patient's ability to engage in specialized communication (52). If the patient fails to meet this expectation, this may cause the doctor to form impressions about the patient, for example, perceiving them as difficult to communicate with or excessively anxious (52). The doctor–patient trust crisis can then become more severe. The behavior of seeking health information online is related to several factors. People with higher educational and health literacy levels, as well as those in developed countries compared to developing countries, are more likely to engage in online health information-seeking (53). The concentration in megacities of populations with a higher educational level and superior health literacy, exemplified by Beijing's leading rates in both metrics nationwide (see Section 3.1), coupled with their generally developed socioeconomic contexts, leads to a higher prevalence of online health information seeking, which in turn predisposes these urban centers to an exacerbated doctor–patient trust crisis.

The use of artificial intelligence, while enhancing patient autonomy, may therefore also intensify conflicts between patients and doctors. Increased patient autonomy enables individuals to participate more actively in medical decision-making. However, the current application of artificial intelligence in healthcare is often constrained by algorithmic discrimination and opacity, which can lead to misdiagnosis and disputes. Similarly, tensions may arise when patients use artificial intelligence- generated recommendations to challenge doctors’ treatment plans. In cases involving artificial intelligence in medical processes, the lack of clear regulations on liability allocation in medical disputes further exacerbates conflicts. Moreover, as a patient's autonomy increases, they grow increasingly concerned about personal privacy protection. The use of artificial intelligence, which relies heavily on large-scale data, requires a careful balance between technological application and privacy safeguards, which is crucial to maintaining a good doctor–patient relationship. In megacities, patients often have high expectations regarding treatment outcomes. However, high patient volumes frequently lead to scenarios where hours of waiting result in only brief consultations. When patients have already used artificial intelligence to research their conditions or who have formed preconceived diagnoses and then receive only limited time with a doctor to receive explanations, misunderstandings and disagreements are more likely to occur. Furthermore, megacities’ technological advantages mean that their development and implementation of artificial intelligence in healthcare are more advanced, involving larger volumes of data. This, in turn, increases the potential risks to patient privacy, adding another layer of complexity to the doctor–patient relationship.

The insertion of artificial intelligence as a third actor in the traditional doctor–patient dualistic interaction structure reconstitutes it into a technologically mediated triad interaction structure that inherently increases physical and psychological distance. Doctors should put themselves in the patients’ shoes and gain a comprehensive understanding of them before treating the disease accordingly. The integration of artificial intelligence reduces the necessity for doctors and patients to be in the same room. While enhancing efficiency, this displaces the interaction between doctors and patients, which may make it feel cold and impersonal. Additionally, the interaction focus shifts from the doctor–patient to doctor–artificial intelligence and patient–artificial intelligence, resulting in a decrease in empathy. While one study showed that the application of artificial intelligence could save doctors time, allowing them to spend more time communicating with the patient (54), no research has proved that the time saved by artificial intelligence is actually used for communication between doctors and patients. On the contrary, due to the burden on the healthcare system in megacities such as Beijing, the time saved by artificial intelligence may be used to see more patients (54). This means that doctors may still not have enough time for full and effective communication with each patient, leading to a deterioration in the emotional relationship between doctors and patients (54).

## 5 Suggestions for doctor–patient relationship governance in megacities in the artificial intelligence era

While the application of artificial intelligence in healthcare demonstrates considerable potential to enhance the quality of healthcare services, it also exerts an inevitable influence on the doctor–patient relationship. These effects are further amplified within the unique socio-technical complexity of megacities. In the era of artificial intelligence, proactive governance is essential to better harness its benefits while mitigating its risks.

### 5.1 Institutionalizing the doctor–artificial intelligence–patient triad interaction structure

The application of artificial intelligence in healthcare has transformed the traditional doctor–patient dualistic interaction structure into a doctor–artificial intelligence–patient triad interaction structure. This paradigmatic shift is distinctly intensified in megacities (see Section 3), where the adoption of artificial intelligence in healthcare is large-scale and fast-paced. Therefore, it is important to institutionalize the doctor–artificial intelligence–patient triad interaction structure in megacities. In this triad interaction structure, the doctor has to preserve the primary role in final decision-making, while artificial intelligence plays a supporting role in improving diagnostic accuracy and reducing doctors’ workload. Concurrently, preserving doctors’ primacy in final decision-making can prevent liability ambiguities arising from algorithmic discrimination and opacity.

### 5.2 Leveraging megacity advantages for the application of artificial intelligence in healthcare

Megacities usually attract large numbers of high-tech talent and innovative enterprises. These cities must therefore leverage their concentrated technological capital to pioneer the integration of artificial intelligence and healthcare. Strategic development should first target the challenges that megacities face, such as patient volume pressures, disproportionate share of premium medical resources, and so on, to enhance healthcare services. Improving artificial intelligence's capacity in this area could de-escalate relational risk factors in the doctor–patient relationship and promote its harmonious development.

### 5.3 Policy-driven governance of risks in the application of artificial intelligence in healthcare

Given their scale and advantages, when megacities develop and apply artificial intelligence in healthcare, they should enact preemptive governance frameworks to mitigate and regulate the associated risks and drawbacks. First, corresponding laws and regulations should be passed to provide clear provisions on patient privacy protection and determining liability in medical disputes. Second, corresponding ethical standards should be established to prioritize patient interests while avoiding ethical dilemmas for medical professionals. Finally, regulatory authorities should strengthen oversight, conduct comprehensive assessments of the potential impact of artificial intelligence on doctor–patient relationships, and intervene promptly to actively adjust these relationships.

## 6 Conclusion

As Tedros Adhanom Ghebreyesus, the WHO director-general, stated, “artificial intelligence is already playing a role in diagnosis and clinical care, drug development, disease surveillance, outbreak response, and health systems management… The future of healthcare is digital, and we must do what we can to promote universal access to these innovations and prevent them from becoming another driver for inequity.” (55). The integration of artificial intelligence and healthcare could bridge information asymmetry, advance patient autonomy, and transform the doctor–patient dualistic interaction structure into a doctor–artificial intelligence–patient triad interaction structure. However, it could also inadvertently exacerbate the doctor–patient trust crisis, intensify conflicts, and increase physical and psychological distance between patients and doctors. The socio-technical complexity of megacities amplifies these effects. Rather than rejecting the adoption of artificial intelligence, megacities such as Beijing should adapt their governance frameworks to better harness its benefits while mitigating its associated risks.

## Data Availability

The original contributions presented in the study are included in the article/Supplementary Material, further inquiries can be directed to the corresponding author.
